# The hemostatic and comforting effects of oral adhesive bandages in tooth extraction: a randomized controlled clinical study

**DOI:** 10.1007/s00784-024-05648-9

**Published:** 2024-04-07

**Authors:** Xiaocheng Zhou, Yu Cai, Jihong Zhao

**Affiliations:** 1 Department of Oral and Maxillofacial Surgery, School & Hospital of Stomatology, Wuhan University, Wuhan, China; 2 The State Key Laboratory Breeding Base of Basic Science of Stomatology, Hubei Province & Key Laboratory of Oral Biomedicine, Ministry of Education, Wuhan University, Wuhan, China

**Keywords:** Oral adhesive bandages, Tooth extraction, Hemostasis, Comfort, Healing

## Abstract

**Objectives:**

To compare oral adhesive bandages with the classic compression method and evaluate the clinical efficacy of this wound dressing material in improving postoperative comfort, wound healing, and hemostasis in tooth extraction.

**Materials and methods:**

The study was designed as a randomized controlled clinical trial. A total of 120 patients were recruited and randomly assigned to the study group and the control group. In the study group, oral adhesive bandages were used as wound dressing. In the control group, patients bit on cotton balls and gauze, as usual. Hemorrhage, comfort, and healing levels were evaluated at postoperative 1 h, 24 h, and 7 days. The adhesion time of the oral adhesive bandages was also recorded.

**Results:**

The average adhesion time of the oral adhesive bandages was 26.6 h. At postoperative 1 and 24 h, the hemostatic levels of the oral adhesive bandage group were significantly higher than those of the control group. The oral adhesive bandage group also reported significantly higher comfort scores than the control group. Both groups had similar healing levels and side effects. But the mean score for wound healing was slightly higher in the oral adhesive bandage group.

**Conclusions:**

Oral adhesive bandages were more effective than cotton balls and gauze in providing hemostatic and comfort effects on extraction wounds.

**Clinical relevance:**

Oral adhesive bandages possess clinical value in the management of extraction wounds.

## Introduction

Tooth extraction is a common operation in oral and maxillofacial surgery. Several strategies have been widely used for hemostasis in tooth extraction, including compression, packing, and suture. However, these methods have their own limitations. Specifically, although compression with cotton balls and gauze is economical and convenient, patients usually have to bite on the cotton balls and gauze for 30 to 60 min. During this period, speaking, chewing, and drinking are forbidden to maintain the position of the cotton balls and gauze. Continuous jaw clenching can cause significant discomfort for patients. After removal of the cotton balls, the sockets are exposed to the oral cavity, and actions such as spitting, sucking, eating, and rinsing can disturb the blood clots within the sockets, leading to potential bleeding. Lacking coverings that prevent irritants from entering the sockets may elevate the risks of dry socket, infection, and delayed wound healing. Packing with hemostatics and suturing usually requires a combination with the compression method. Additionally, inserts like gelatin and collagen sponges, as well as hemostatic agents (e.g., oxidized regenerated cellulose, synthetic hyaluronic acid, hydrogel), and threads placed in the extraction sockets may increase the risks of inflammation, infection, and pain [1–3]. Therefore, better protection methods and materials for extraction wound management deserve to be developed.

The oral adhesive bandages are a type of wound dressing material that can adhere to wounded areas and durably shield the wounds from local stimuli in the oral cavity. Since their first introduction in 1968, different ingredients and compositions of oral adhesive bandages have been reported, such as the extracellular matrix-mimicking hydrogel, gelatin-sodium carboxymethylcellulose-polyisobutylene complex, and biotinylated polyacrylic acid [4, 5]. Several clinical trials have been conducted to explore the characteristics of oral adhesive bandages in various dental practices, including tooth extraction. According to the findings of these trials, most bandages adhered to the wounds for less than 24 h; only half to two-thirds of them achieved marked hemostatic and pro-healing effects; some patients still reported poor tolerance to the bandages [6]. For extraction sockets, continuous and durable isolation from irritants is critical for hemostasis and the healing of wounds. New types of oral adhesive bandages that can achieve long duration of adhesion, provide excellent hemostatic and pro-healing effects, and offer a comfortable experience, would provide benefits for patients and deserve to be explored. In this clinical trial, we chose a new type of oral adhesive bandage and compared it with traditional compression materials (cotton balls and gauze) to evaluate whether this wound dressing material could provide durable isolation, ideal hemostasis and protection, and a comfortable experience following tooth extraction.

## Materials and methods

### Study design and patient recruitment

The study was designed as a randomized controlled clinical trial and approved by the medical ethics committee in the Hospital of Stomatology, Wuhan University (NO. 2022-C12). The whole study was organized following the Consolidated Standards of Reporting Trials statement and all procedures were conducted in accordance with the 1964 Declaration of Helsinki. Written informed consent was obtained from all participants.

The study group and the control group were set up in this trial. The sample size was estimated by using a calculator from PowerAndSampleSize.com with 0.8 power to detect statistical differences between the groups. The type I error rate and dropout rate were set at 5% and 20%, respectively. The sample size was set at *n* = 60/group.

Inclusion criteria were: patients aged 18–65 years who required surgical removal of one single tooth (except upper and lower third molars) at a time. Exclusion criteria were as follows: menstruation, pregnancy or lactation, periodontitis, tobacco usage, coagulation disorders, infection or immune dysfunction, a history of allergy to any drugs, cyst or tumor, and unwillingness to participate in this trial. Blood tests were performed routinely to exclude patients who suffered from coagulation disorders or any serious hematological diseases.

Patients who met the inclusion criteria were randomly divided into the study and control groups by the lottery method. In the study group, oral adhesive bandages were applied to the gingiva and covered the sockets immediately after tooth extraction, remaining in place until they naturally fell off. In the control group, patients bit on cotton balls and gauze for 1 h.

### Surgery

The same dentist performed tooth extraction surgeries for all patients following the standard procedure. The surgical area was prepared with 5% povidone iodine solution. All patients were given local anesthesia. The teeth were removed by using dental elevators or forceps, and curettage of the sockets was performed. Subsequently, different hemostasis materials were placed onto the sockets: oral adhesive bandages for the study group, cotton balls and gauze for the control group.

### Oral adhesive bandage taping

**Fig. 1:**
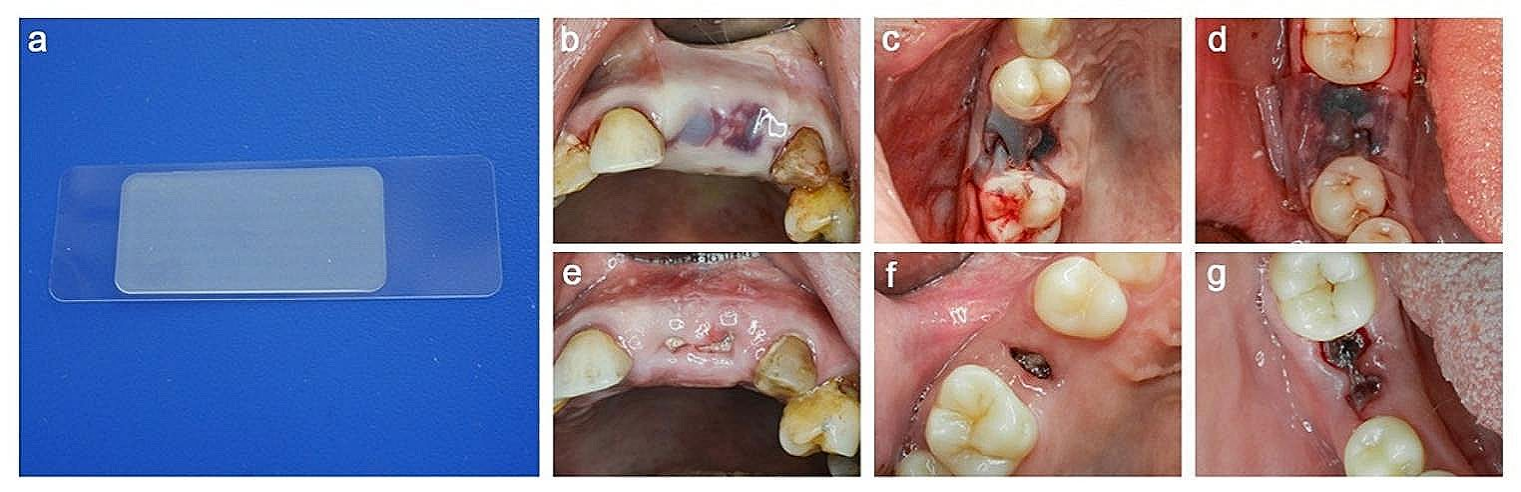
Application of the oral adhesive bandages on extraction sockets. (**a**) Photo of the oral adhesive bandage. (**b**, **c**, **d**) Application of the oral adhesive bandages after tooth extraction. (**e**, **f**, **g**) 7 days after extraction surgeries

The oral adhesive bandage (BONANGA, CHINA) we chose is composed of an absorbable adhesive layer and a nonabsorbable shielding layer (Fig. 1). The biodegradable adhesive layer is made from hydroxyethyl cellulose, polyvinylpyrrolidone, and corrigent. The nonabsorbable shielding layer is made from ethyl cellulose. The oral adhesive bandage is underlaid by a removable polyethylene film. This type of oral adhesive bandages has two subtypes: type A is designed as a rectangular shape (length: 30 mm, width: 15 mm, thickness: 0.4 mm), while type C is a shorter rectangular shape with a small concave arc on each long side (length: 25 mm, width: 15 mm, thickness: 0.4 mm). This type of oral adhesive bandage has been approved by the Chinese Food and Drug Administration for oral use (approval number: 20,200,006). In this trial, we chose type A oral adhesive bandages.

When teeth were extracted, blood and saliva on the mucosal surface around the extraction socket were wiped out with cotton balls and saliva suction tubes. The dentist tore the oral adhesive bandages off the polyethylene films and stuck the adhesive side of the bandages to the gingival surface around the sockets. To ensure adhesive strength, continuous pressure on the bandages for over 15 s was required. Then the oral adhesive bandages would stick to the extraction sockets and tightly cover the holes to protect the blood clots. As the absorbable adhesive layer gradually dissolved, the shielding layer would automatically peel off the sockets (Fig. 1).

### Assessment and data collection

At postoperative 1 and 24 h, the dentist examined the extraction sockets, evaluated and scored the bleeding based on the following standard: 0, severe bleeding, requiring hemostatic measures; 1, moderate bleeding; 2, slight bleeding; 3, no postsurgical bleeding. At postoperative 1 h, all patients were given a comfort rating scale which ranges from 0 to 10 (0, severe discomfort; 1 ~ 3, slight discomfort; 4 ~ 6, normal; 7 ~ 9, slight comfort; 10, high comfort), and scored the comfort levels for the method they used. On postoperative day 7, another dentist that working under a single-blind principle (unaware of which group patients belonged to) examined all patients and evaluated the healing of wounds following such a standard: 0, moderate to severe tenderness or pain, moderate to severe inflammation reaction in the surgical area; 1, slight to moderate swelling and tenderness, slight reddened; 2, no swelling and tenderness, normal appearance.

Besides, the adhesive time of all oral adhesive bandages and any adverse reactions were recorded.

### Statistical analysis

Data were recorded by Microsoft Excel and analyzed by Statistical Product and Service Solutions (SPSS) 21 software (SPSS Inc., Chicago, USA). Clinically evaluated items were analyzed by the Mann-Whitney U test and the Student’s t-test. Results were considered statistically significant when *P* < 0.05.

## Results

We recruited 120 patients (49 males and 71 females) and randomized them into the study group and the control group. Both groups had similar demographic characteristics (Table 1). The adhesion time of oral adhesive bandages in the study group is presented in Table 2. The clinical data revealed that the average adhesion time of oral adhesive bandages was 26.6 h. 40% of the bandages remained in place on the extraction sockets for an average of 5.7 h, while 28% lasted for an average of 21.3 h. Additionally, there were 13% and 4% of bandages remaining in place for 30.7 h and 45.5 h, respectively. Furthermore, 15% of bandages remained at the sites of placement for 83.4 h (Table 2).

The clinical data, including the scores of hemostasis, healing, comfort, and side effects, is presented in Table 3. At postoperative 1 and 24 h, the hemostatic scores of the oral adhesive bandage group, which were 2.88 and 2.97, were significantly higher than those of the cotton balls and gauze group, which were 2.40 and 2.88, respectively (Table 3). The oral adhesive bandage group also scored significantly higher in terms of comfort compared to the control group (Table 3). Both groups had similar healing effects and side effects, but the mean score of the oral adhesive bandage group was slightly higher than that of the control group. The adverse event in the oral adhesive bandage group was a case of slight nausea. After the bandage was removed, the nausea obviously subsided.

**Table 1:** Demographic characteristics and tooth extraction information of participants

	Study group (Oral adhesive bandages, *n* = 60)	Control group (Cotton balls and gauze, *n* = 60)	*p*
Gender			0.353^a^
Men	22 (36.7%)	27 (45%)	
Women	38 (63.3%)	33 (55%)	
Age (years)			0.149^b^
Range	18–59	18–62	
Mean ± SD	29.8 ± 12.1	33.2 ± 13.3	
Tooth position			0.107^c^
Range	1–7	1–7	
Mean	4.8	5.1	
Duration of extraction (min)			0.190^d^
Range	2–15	2–15	
Mean ± SD	3.6 ± 2.3	4.1 ± 2.5	

^a^p-value for the comparison of gender distribution between groups (χ^2^ test)

^b, c, d^p-value for the comparison of age distribution, tooth number, and extraction time between groups (T test)

**Table 2:** Adhesion time of oral adhesive bandages in participants

Adhesion time (hours)	Number of teeth	Percentage	Mean (hours)
0–12	40	40%	5.7
13–24	28	28%	21.3
25–36	13	13%	30.7
37–48	4	4%	45.5
49 or more	15	15%	83.4
Total	100	100%	
	Mean of adhesion time (hours)		26.6

**Table 3:** Scores of hemostasis, healing, comfort, and side effects in all participants

	Study group (Oral adhesive bandages)	Control group (Cotton balls and gauze)	*p*
Hemostasis			
Postoperative 1 h	2.88	2.40	< 0.0001^e^
Postoperative 24 h	2.97	2.88	0.02^e^
Healing			
Postoperative 7 days	1.99	1.97	0.42^f^
Comfort			
Postoperative 1 h	8.40	4.57	< 0.0001^g^
Side effects	1	0	0.32^h^

^e, f, g, h^p-value for the comparison of hemostatic, healing, comfort levels, and side effects between groups (T test)

## Discussion

Several strategies and materials, including compression, packing, suture, and oral adhesive bandages, have been applied in extraction wound management. The oral adhesive bandages can isolate the extraction sockets from the wet and dynamic environment of the oral cavity, and promote the healing of wounds. However, according to the findings of previous studies, the adhesion time of most oral adhesive bandages was less than 24 h. Moreover, the hemostatic, pro-healing, and comfort effects of these bandages were still far from ideal. Therefore, new types of oral adhesive bandages that can maintain long duration of adhesion, provide excellent hemostatic and pro-healing effects, and offer a superior comfort experience would greatly benefit patients and warrant further exploration. In this clinical trial, we have chosen a novel type of oral adhesive bandage and compared it with traditional compression materials (cotton balls and gauze) to evaluate its ability to provide durable isolation, ideal hemostasis and protection, as well as a positive comfort experience following tooth extraction. The results of our study indicated that the mean adhesion time of this new type of oral adhesive bandage was 26.6 h. Compared to the standard compression method, the oral adhesive bandages provided significantly better hemostatic effects, and offered marked comfort experiences. Moreover, this wound dressing material achieved higher scores of wound healing than the traditional compression material. These results indicate that this new type of oral adhesive bandage is effective in extraction wound management and has great potential for dental practices.

Just like adhesive wound plasters which were widely used in skin wounds, oral adhesive bandages were first reported in 1968. They could provide mechanical and chemical protection for wounds of oral cavity [7–9]. Early reports indicated that oral adhesive bandages could be applied in tooth extraction, but the actual effects of the bandages on extraction wound management were still far from ideal [6, 8]. In this clinical trial, we chose a novel type of oral adhesive bandage. Our results revealed that the mean adhesion time of this new type of oral adhesive bandage was 26.6 h (Table 2) which was longer than the protection time of standard compression method and the critical period of blood clot formation and stabilization. In previous studies, the adhesion time of most oral adhesive bandages was less than 24 h [6]. Unlike previous types, the new type of oral adhesive bandages consists of two layers including an absorbable adhesive layer and a nonabsorbable protecting layer. Possessing robust adhesive strength, the adhesive layer can firmly stick on the moist gingiva. After the adhesive layer gradually dissolves, the protecting layer can continuously remain in place on the sites of application and effectively isolate the sockets from irritants. Moreover, the size of this new type of oral adhesive bandage guarantees the complete sealing of extraction sockets and the coagulation of blood clots. Therefore, this novel type of oral adhesive bandages can provide significantly better hemostatic effect than the traditional compression method. Similarly, previous study showed that the oral adhesive bandages could provide marked or moderate hemostatic effect in many extraction cases. Additionally, the new type of oral adhesive bandages only requires 15 s of pressure to initiate adhesion, while the oral adhesive bandages reported in previous study require at least 1 min of pressure to initiate adhesion [6]. Therefore, this new type of oral adhesive bandages possesses robust adhesive property and can provide durable protection and an excellent hemostatic effect for extraction sockets. For patients on anticoagulation, the combined application of this new type of oral adhesive bandages, packing, and sutures after tooth extraction might achieve effective hemostasis and deserves further exploration.

What is noteworthy is that the mean comfort score of the oral adhesive bandage group was significantly higher than the control group (Table 3). The new type of oral adhesive bandage is a piece of rectangular sheet at merely 0.4 mm thick. It is imperceptible when this type of oral adhesive bandages smoothly covers the extraction socket. Patients do not have to bite on cotton balls and gauze. They can talk, drink, and even eat soon after extraction surgeries. They can also brush teeth and rinse mouths on the day of the surgeries. Furthermore, this type of oral adhesive bandages can continuously protect the wounds from local stimuli that often cause discomfort and pain. Therefore, consistent with previous studies, this wound dressing material offers a superior comfort experience.

Although there was no statistical difference between both methods in wound healing scores, the mean score of the oral adhesive bandage group was higher than that of the control group (Table 3). This result indicates that extended duration of isolation can protect the extraction sockets from stimuli and promote the healing of extraction wounds. Previous study showed that the oral adhesive bandages could promote half of the oral surgical wounds healing faster than the unbandaged wounds [6]. Another study found that covering wounds with autologous fibrin glue and polyglycolic acid sheets can provide long-term protection and significantly promote the healing of wounds [10]. In our study, the average 26.6 h of protection was still too short for the healing of extraction wounds. Therefore, further research is needed to upgrade the ingredients and compositions of oral adhesive bandages to achieve longer protection time and better pro-healing effects.

However, there are some challenges we have to face. The case of slight nausea that occurred in the oral adhesive bandage group reminds us that there are some risks of complications towards this wound dressing. The polyvinylpyrrolidone, one of the ingredients of the oral adhesive bandages, has been verified as an allergen [11, 12]. Patients who are too young or have allergic problems might not be suitable to this wound dressing material. Moreover, the adhesion procedure of the oral adhesive bandages is more difficult than the traditional compression method. If dentists cannot initially stick the bandages on the gingiva, saliva and blood would contaminate the adhesive sides of the bandages and weaken the adhesive strength of them. Furthermore, the price of the oral adhesive bandages is higher than suture, cotton balls, and gelatin sponges. It is necessary to lower the cost of the oral adhesive bandages for extended application. Therefore, the new type of oral adhesive bandages still need further upgrades for extraction wound management.

## Conclusions

The oral adhesive bandages are more effective than the traditional compression method in hemostatic and comforting effects on extraction wounds, and exhibit high clinical value in extraction wound management.

## Data Availability

No datasets were generated or analysed during the current study. Clinical study registration number: ISRCTN11589028; date of registration: 20th November, 2023.
